# Common Distribution of *gad* Operon in *Lactobacillus brevis* and its GadA Contributes to Efficient GABA Synthesis toward Cytosolic Near-Neutral pH

**DOI:** 10.3389/fmicb.2017.00206

**Published:** 2017-02-14

**Authors:** Qinglong Wu, Hein Min Tun, Yee-Song Law, Ehsan Khafipour, Nagendra P. Shah

**Affiliations:** ^1^School of Biological Sciences, The University of Hong KongHong Kong, Hong Kong; ^2^Department of Animal Science, University of ManitobaWinnipeg, MB, Canada; ^3^Department of Medical Microbiology, University of ManitobaWinnipeg, MB, Canada; ^4^Victoria UniversityMelbourne, VIC, Australia

**Keywords:** genomic survey, *Lactobacillus brevis*, γ-aminobutyric acid (GABA), glutamic acid decarboxylase, acid resistance

## Abstract

Many strains of lactic acid bacteria (LAB) and bifidobacteria have exhibited strain-specific capacity to produce γ-aminobutyric acid (GABA) via their glutamic acid decarboxylase (GAD) system, which is one of amino acid-dependent acid resistance (AR) systems in bacteria. However, the linkage between bacterial AR and GABA production capacity has not been well established. Meanwhile, limited evidence has been provided to the global diversity of GABA-producing LAB and bifidobacteria, and their mechanisms of efficient GABA synthesis. In this study, genomic survey identified common distribution of *gad* operon-encoded GAD system in *Lactobacillus brevis* for its GABA production among varying species of LAB and bifidobacteria. Importantly, among four commonly distributed amino acid-dependent AR systems in *Lb. brevis*, its GAD system was a major contributor to maintain cytosolic pH homeostasis by consuming protons via GABA synthesis. This highlights that *Lb. brevis* applies GAD system as the main strategy against extracellular and intracellular acidification demonstrating its high capacity of GABA production. In addition, the abundant GadA retained its activity toward near-neutral pH (pH 5.5–6.5) of cytosolic acidity thus contributing to efficient GABA synthesis in *Lb. brevis*. This is the first global report illustrating species-specific characteristic and mechanism of efficient GABA synthesis in *Lb. brevis*.

## Introduction

Many species of lactic acid bacteria (LAB) and bifidobacteria are of economic and health importance and have been extensively used for the production of yogurt, cheese, milk beverage, and fermented vegetables for thousands of years (Leroy and De Vuyst, 2004). These bacteria have been considered as potential probiotics for promoting human health (Hill et al., 2014). Interactions between ingested LAB or bifidobacteria and host are associated with immunomodulation (Bron et al., 2012; van Baarlen et al., 2013; Sivan et al., 2015), gut homeostasis maintenance (Gareau et al., 2010; Delzenne et al., 2011), and brain behavior (Tillisch et al., 2013; Möhle et al., 2016). Moreover, metabolites from these bacteria, such as lactate, have also been extensively evaluated for brain function and plasticity promotion (Herzog et al., 2013; Tang et al., 2014; Yang et al., 2014), bacteriocin for killing pathogens (Cotter et al., 2013; Martinez et al., 2013), exopolysaccharide as food texturing agent (Wu et al., 2014) and immune modulator (Fanning et al., 2012). In the past decades, γ-aminobutyric acid (GABA) has drawn a lot of attentions because GABA is the most abundant inhibitory neurotransmitter that maintains neuro functions of human central nervous system. Although GABA may not be able to cross human blood-brain barrier (Kuriyama and Sze, 1971; Boonstra et al., 2015), GABA approved as a food ingredient and its food carriers have shown anti-hypertensive and anti-depressant activities as two main functions to the host after oral administration (Diana et al., 2014; Wu and Shah, 2016). However, GABA content in natural animal and plant products is very low, thus solutions have been sought from microorganisms including LAB and bifidobacteria for their capability of producing GABA.

GABA synthesis via glutamate decarboxylation in bacteria has been associated with acid resistance (Su et al., 2011; De Biase and Pennacchietti, 2012; Teixeira et al., 2014). Glutamic acid decarboxylase (GAD) system encoded by the *gad* operon is responsible for glutamate decarboxylation and GABA secretion in bacteria and consists of two important elements—Glu/GABA antiporter GadC and GAD enzyme either GadA or GadB (Capitani et al., 2003). This system converts glutamate into GABA and while doing so consumes protons thus maintaining cytosolic pH homeostasis (Krulwich et al., 2011). Our recent review and another previous review summarized enormous reports presenting specific strains of LAB and bifidobacteria having varying capacities to produce GABA (Li and Cao, 2010; Wu and Shah, 2016). This suggests that the efficiency of GAD system in these bacteria depends on their activities of Glu/GABA antiporter and GAD enzyme. Thus, most studies have been carried out to characterize individual strains for their GABA production in varying conditions either controlled or natural fermentations (Li and Cao, 2010; Dhakal et al., 2012; Wu and Shah, 2015, 2016). Among these reported strains, we found that isolates of *Lactobacillus brevis* appear to be efficient for GABA production (Wu and Shah, 2016). Importantly, these GABA-producing *Lb. brevis* strains have different sources of origin such as human intestine, Korean kimchi and brewery, suggesting their varying genetic backgrounds but exhibiting similar GABA synthesis capacities (Wu and Shah, 2016).

Currently, hundreds of species of LAB and bifidobacteria that have been systematically identified, and their genome sequences have been deposited in public databases including DDBJ/EMBL/GenBank databases. Due to the importance and applications of LAB and bifidobacteria in food industry and human health, it is crucial to gain insights into global diversity of GABA-producing bifidobacteria and LAB mainly including *Lactobacillus* and *Lactococcus*. These bacteria are able to catabolize sugars to produce lactic acid efficiently (Leroy and De Vuyst, 2004), but they have also developed multiple acid resistance systems including F_0_F_1_-ATPase proton pump, sodium/proton antiporter, amino acid decarboxylation and deimination, alkali production and biofilm formation (Hutkins and Nannen, 1993; Zhao and Houry, 2010; Liu et al., 2015). Since multiple strategies as stated above in these bacteria are available to cope against acids, the linkage between bacterial acid resistance and GABA production capacity derived from GAD system has not been well established. Moreover, it is vital to unravel the mechanism of efficient GABA synthesis in these isolates especially in *Lb. brevis*. In the present study, *Lb. brevis* NPS-QW-145 (hereafter *Lb. brevis* 145) isolated previously from Korean kimchi was used as a model organism of high GABA-producing lactic acid bacterium (Wu and Shah, 2015). The chromosome of this organism has been completely sequenced in this study; genomic survey and biochemical toolkits have been carried out to illustrate efficient machinery of GABA production from this organism.

## Materials and methods

### Bacterial strains, cultivation conditions, and genome sequencing

GABA-producing *Lb. brevis* 145 and a reference strain, *Lb. plantarum* WCFS1, were cultivated in Lactobacilli MRS medium (BD Company, Franklin Lakes, NJ). Unless otherwise stated, *Lactobacillus* strains used in this study were anaerobically cultivated in the above medium statically. For cloning and protein expression purposes, *E. coli* strains as indicated in Table 1 were cultivated in Luria-Bertani (LB) medium with or without antibiotics supplemented to the medium. The model strain of high GABA-producing *Lb. brevis* 145 was completely sequenced in this study (please refer to Supplementary Materials and Methods for details). The NCBI accession number for the chromosome of *Lb. brevis* NPS-QW-145 is CP015398.

Table 1**Bacterial strains, plasmids and primers used in this study**.

**Table:** 

**Bacterial strains**		
**Species**	**Strain ID**	**Description**
*Lactobacillus brevis*	NPS-QW-145	High GABA producer carrying a *gadB* and a *gadA* in an intact *gad* operon
*Lactobacillus plantarum*	WCFS1	Negative GABA producer carrying *gadB* only as a reference
*Escherichia coli*	DH5α	Cloning host
*Escherichia coli*	XL1-Blue	Cloning host
*Escherichia coli*	BL21(DE3)pLysS	Expression host
*Escherichia coli*	BL21(DE3)	Expression host
**Plasmids**		
**ID**	**Antibiotic resistance**	**Description**
pCXSN	Kanamycin	Cloning vector
pET-28a(+)	Kanamycin	Expression vector
pRSETA-SUMO	Ampicillin	In-house expression plasmid based on pRSET-A (Invitrogen) with an N-terminal His-SUMO tag
pET-28a(+)-Lb-gadA	Kanamycin	Expression plasmid carrying *gadA* (+GSHM) from *Lb. brevis* NPS-QW-145
pET-28a(+)-Lb-gadB	Kanamycin	Expression plasmid carrying *gadB* (+GSHM) from *Lb. brevis* NPS-QW-145
pET-28a(+)-Lp-gadB	Kanamycin	Expression plasmid carrying *gadB* (+GSHM) from *Lb. plantarum* WCFS1
pRSETA-SUMO-Lb-gadA	Ampicillin	Expression plasmid carrying *gadA* (WT) from *Lb. brevis* NPS-QW-145
pRSETA-SUMO-Lb-gadA Δ5	Ampicillin	Expression plasmid carrying *gadA* mutant (Δ5) from *Lb. brevis* NPS-QW-145

**Table:** 

**Primers**				
**Primer ID**	**Sequences (5′ → 3′)**	**Amplicon size (bp)**	**Target gene**	**Reference**
**PRIMERS FOR PROTEIN HETERO-EXPRESSION**				
Lb-gadA-NdeI-F	tataCATATGatgaataaaaacgatcaggaaac	1462 or 1460 or 1447	*gadA* in *Lb. brevis* NPS-QW-145	This study
Lb-gadA-AgeI-F	attACCGGTGGAatgaataaaaacgatcaggaaac			
Lb-gadA Δ5-AgeI-F	attACCGGTGGAcaggaaacacagcagatgattaat			
Lb-gadA-HindIII-R	gcatAAGCTTttaacttcgaacggtggtc			
Lb-gadB-NdeI-F	gccgCATATGatggctatgttgtatgg	1424	*gadB* in *Lb. brevis* NPS-QW-145	This study
Lb-gadB-HindIII-R	gcggAAGCTTttagtgcgtgaacccgtatt			
Lp-gadB-NdeI-F	tgcgCATATGatggcaatgttatacggtaaacac	1427	*gadB* in *Lb. plantarum* WCFS1	This study
Lp-gadB-EcoRI-R	agctGAATTCtcagtgtgtgaatccgtatttc			
**PRIMERS FOR REAL-TIME qPCR ASSAY**				
Lb-tuf-F	CGTGAGCTCTTGTCTGAATAC	152	*tuf* in *Lb. brevis* NPS-QW-145 (reference gene)	Schurr et al., 2013
Lb-tuf-R	CGTTCTGGAGTTGGGATATAAT			
Lb-PTC-F	GCCAGAAACGCTCAAGAT	16	*aguB* in AgDI pathway of *Lb. brevis* NPS-QW-145	This study
Lb-PTC-R	GGCTTCGTATAAGCCATACC			
Lb-OTC-F	GTGAAAGCAACTGGGAAGA	128	*arcB* in ADI pathway of *Lb. brevis* NPS-QW-145	This study
Lb-OTC-R	GTTATGGAAAGCAGGCAAAC			
Lb-TDC-F	CGATCAAGCAGAGTCCATTAC	140	*tyrDC* in TDC pathway of *Lb. brevis* NPS-QW-145	This study
Lb-TDC-R	CGGCACCCTTCTCAAATAC			
Lb-gadA-757F	CAGGTTACAAGACGATCATGC	188	*gadA* in GAD pathway of *Lb. brevis* NPS-QW-145	Wu et al., 2015
Lb-gadA-945R	ATACTTAGCCAGCTCGGACTC			
Lb-gadB-364F	GGACAATACGACGACTTAGC	135	*gadB* in GAD pathway of *Lb. brevis* NPS-QW-145	Wu et al., 2015
Lb-gadB-499R	CTTGAGCTCGGGTTCAATAA			
Lp-ldhD-F	ACGCCCAAGCTGATGTTATC	127	*ldhD* in *Lb. plantarum* WCFS1 (reference gene)	Fiocco et al., 2008
Lp-ldhD-R	AGTGTCCCACGAGCAAAGTT			
Lp-gadB-F	GCTCCTCTGAAGCTTGTATG	124	*gadB* in *Lb. plantarum* WCFS1	This study
Lp-gadB-R	TGATAGCCAGCCGAAATAAC			

### Genomic survey on the distribution of *gad* operon and genes encoding glutamate decarboxylase in LAB and bifidobacteria

Currently, there are more than a thousand of strains of LAB and bifidobacteria that have been sequenced and deposited in NCBI-GenBank database. Thus, genomic survey was carried out for the presence or absence of *gad* operon and genes encoding glutamate decarboxylase in most of the sequenced strains (all assembly levels; 890 strains) of *Bifidobacterium* and LAB including *Lactobacillus, Lactococcus, Leuconostoc, Pedicoccus, Oenococcus, Weissella*, and *Streptococcus thermophilus* released in NCBI-GenBank genome database. Only the species having at least two strains sequenced were included in this genomic survey. In addition, the distribution of acid resistance systems including F_0_F_1_-ATPase system, amino acid/cation:proton antiporters, glutamic acid decarboxylase (GAD) system, tyrosine decarboxylase (TDC) system, agmatine deiminase (AgDI) system, arginine deiminase (ADI) system and urea system in *Lb. brevis* was also surveyed in terms of presence or absence of above AR systems in the sequenced strains.

### Extracellular pH (pH_ex_) and intracellular pH (pH_in_) measurements

Extracellular pH (pH_ex_) was measured directly with the pH meter. The fluorescent probe–5(6)-carboxyfluorescein diacetate N-succinimidyl ester (cFDA-SE; Thermo Fisher Scientific) was used to label bacterial cells for intracellular pH (pH_in_) measurement. There are several reports on the pH_in_ measurement of bacteria suspended in citrate-based buffer (Siegumfeldt et al., 2000; Teixeira et al., 2014); however, this buffer did not allow the discrimination between cells equilibrated to pH between 3.5 and 5.0 thus limiting the detection range of cFDA-SE probe (Hansen et al., 2016). Thus, phosphate-buffered saline (PBS) was used to re-suspend bacterial cells for pH_in_ measurement in the range of pH 3.5–7.0 in this study according to the previous studies (Breeuwer et al., 1996; Hansen et al., 2016). Briefly, the pH_ex_ of cultures was measured first, and bacterial cells were centrifuged, washed and re-suspended in PBS where its pH level was adjusted to the pH_ex_ of the cultures. The cell density was adjusted to an optical density (λ = 600) of 0.6–0.7, followed by the addition of both cFDA-SE and glucose to the final concentrations of 10 μM and 10 mM, respectively. The mixture was incubated in the dark at 37°C for 30 min. The stained cells were later harvested by centrifugation at 12,000 × g and 4°C for 5 min, and were re-suspended in the PBS buffer with the same pH_ex_ prior to the measurement.

Fluorescence intensities of the stained cells were measured in the Fluorescence Spectrophotometer F-7000 (Hitachi High-technologies, Shenzhen, China) at the excitation wavelengths of 488 nm (pH-sensitive) and 435 nm (pH-insensitive) by rapidly altering the monochromator between both wavelengths. The emission wavelength was started from 400 to 650 nm. Both excitation and emission slit width was 5 nm. For the calibration curve (pH 3.5–7.0) of *Lb. brevis*, the stained cells were suspended in PBS buffer having different pH values adjusted by hydrochloric acid (HCl). Valinomycin (0.2 mM in methanol; Sigma) and nigericin (0.2 mM in methanol; Sigma) were added to the final concentrations of 5 μM followed by incubation at 37°C for 10 min resulting in the equilibration of both potassium and proton ions across cell membrane. The equilibrated cells were later measure the same as described above.

### Acid resistance and challenge assays

Acid resistance assay was carried out based on previously method described (Seo et al., 2015). Briefly, cells of *Lb. brevis* 145 grown for 12 h (early stationary phase) in Lactobacilli MRS broth were inoculated into Lactobacilli MRS broth (pH 2.5) supplemented with or without 1 g/L of MSG, arginine, agmatine sulfate or tyrosine. The initial cell density after inoculation for acid challenge was 1.4 × 10^8^ CFU/mL. Cells were incubated at 37°C for 2 h statically. Plate count method was then applied to assess the survival rates of *Lb. brevis* cells under above conditions. For acid challenge assay, the 3-h *Lb. brevis* cultures (lag phase, not acid-adapted cells) were centrifuged, washed and suspended in PBS buffer (pH 7.0), followed by cFDA-SE staining as described above. The stained cells were then placed in fluorescence spectrophotometer and their fluorescence emission intensities were measured every 5 min at 37°C. Acid challenge was achieved at the point of 5 min by adding hydrochloric acid that changed pH_ex_ from 7.0 to 3.5. After another 5 min, the stock solution (100 mM) of individual substrate (glutamate, arginine, agmatine, and tyrosine) was added to the final concentration of 10 mM in the solution. The changes in pH_in_ were recorded accordingly as per the above description.

### Growth of *Lb. brevis* 145 in Lactobacilli MRS broth containing arginine, glutamate, agmatine, and tyrosine

Cells of *Lb. brevis* 145 grown for 12 h (early stationary phase) in Lactobacilli MRS broth were inoculated into Lactobacilli MRS broth (pH 6.5) supplemented with or without the mixture of MSG, arginine, agmatine sulfate salt and tyrosine (1 g/L each; Sigma). However, tyrosine was partially dissolved in MRS broth (pH 6.5), and the insoluble tyrosine (solid form in the broth) was removed by membrane filtering (0.45 μm). The initial cell density after inoculation for acid challenge was 1.0 × 10^8^ CFU/mL and the cells were incubated at 37°C for 36 h statically. Samples were collected at the time point of 0, 3, 6, 9, 12, 18, 24, and 36 h. Subsequently, measurements of acids, amino acids, amines and gene expression were carried out as discussed below. Plate count method was used to assess the cell viability, and pH_in_ and pH_ex_ measurements were carried out as stated above.

### Measurements of acids production

Acids in the culture broth were analyzed by HPLC (Model Shimadzu LC-2010A, Shimadzu Corporation, Kyoto, Japan) equipped with Aminex HPX-87H column (300 × 7.8 mm; Bio-Rad). Briefly, cell-free supernatants (diluted if necessary) after removal of bacterial cells and passing through Acrodisc® syringe filter with 0.20 μm Supor® membrane (Pall, Ann Arbor, MI, USA) were injecting into the HPLC system. Acids were eluted with 5 mM sulfuric acid with a flow of 0.60 mL/min. The temperature of the column was maintained at 50°C and the absorbance of the detector was set to 210 nm for detection.

### Measurements of amino acids and amines

Amino acids including glutamic acid, GABA, arginine, ornithine and tyrosine were separated and quantified as per the method we have previously developed (Wu and Shah, 2016). Briefly, cell-free supernatant was collected after centrifugation of fresh cultures. The derivatization was achieved by adding 200 μL acetonitrile, 200 μL NaHCO_3_ (1 M, pH 9.8 adjusted with NaOH), 300 μL distilled water and 100 μL dansyl chloride solution (40 g/L; dissolved in acetonitrile) to 100 μL supernatant (diluted if necessary), then the mixture was kept in an oven (40°C) for 60 min. After derivatization, 100 μL of 20% (v/v) acetic acid was added to stop the reaction and centrifuged at 10,000 × g and 20°C for 2 min. The supernatant was passed through a 0.22 μm membrane filter before HPLC analysis.

Amines including agmatine, putrescine, and tyramine in the supernatants were analyzed as previously described (Dugo et al., 2006). Briefly, 1.6 mL of 10 g/L dansyl chloride solution (dissolved in acetone) was added to 1.5 mL of the supernatant (diluted if necessary). The pH of the mixture was adjusted to pH 8.2–8.3 with 40 g/L of NaCO_3_ solution, then the mixture was heated in a water-bath (40°C) for 60 min in dark. After derivatization, acetone was removed under a stream of nitrogen and then the volume was made up to 5 mL with acetonitrile and centrifuged at 1000 × g and 20°C for 2 min. The supernatant was passed through a 0.22 μm membrane filter before HPLC analysis.

Dansyl amino acids and dansyl amines were separated and detected using a previously developed gradient elution mode (Wu et al., 2015) by HPLC (Model Shimadzu LC-2010A, Shimadzu) equipped with a Kromasil 5 μ 100A C18 column (250 × 4.6 mm; Phenomenex). All the standards of amino acids and amines were derivatized and analyzed as the same in the above procedures. The absorbance for dansyl-amino acids and dansyl-amines were recorded at 275 nm and 254 nm, respectively.

### Total RNA extraction and real-time quantitative PCR assay

Total RNA extraction using hot SDS/phenol method, DNase I treatment, cDNA synthesis, qPCR assay were carried out as per previously described (Wu et al., 2015). The expression of target genes (*gadA, gadB, aguB, arcB*, and *tyrDC* in *Lb. brevis* 145; *gadB* in *Lb. plantarum* WCFS1) listed in Table 1 was quantified by real-time qPCR assay. Reference genes including *tuf* in *Lb. brevis* and *ldhD* in *Lb. plantarum* listed in Table 1 were used to normalize the expression of target genes in both strains. The efficacy of qPCR amplification using each pair of primers was in the range of 90–110%. Comparative critical threshold method (2^−ΔΔCt^) was used to calculate the relative gene expression. The qPCR assay was performed in duplicates for each sample and independent experiments were carried out in triplicates.

### Glutamate decarboxylation assay

For the procedure of cloning, hetero-expression and purification of GadA and GadB from *Lb. brevis* 145 and GadB from *Lb. plantarum* WCFS1, please refer to Supplementary Materials and Methods for details. The effects of pH (3.0–6.6) and temperature (30–90°C) on the purified GADs including *Lb. plantarum* GadB, *Lb. brevis* GadB, and *Lb. brevis* GadA and its mutants (*Lb. brevis* GadA Δ5 and *Lb. brevis* GadA +GSHM) were carried out. Low concentrations of PLP such as 20 μM is sufficient to activate the activities of both *Lb. plantarum* GadB and *Lb. brevis* GadB (Fan et al., 2012; Yu et al., 2012; Shin et al., 2014), thus the reaction mixture consisted of 5 μL of 1 M monosodium glutamate (MSG; Sigma), 5 μL of 2 mM pyridoxal 5′-phosphate (PLP; Sigma), 500 μL of McIlvaine (citrate-phosphate) buffer, and 10 μg of GAD. Previous studies have indicated the PLP- and sulfate ion-dependent activation of *Lb. brevis* GadA and its homologous *Lb. zymae* GadB (Ueno et al., 1997; Park et al., 2014); thus, for *Lb. brevis* GadA and its mutants, the reaction mixture contained 5 μL of 1 M MSG, 5 μL of 20 mM PLP, 500 μL of sulfate buffer (0.8 M sodium sulfate and 50 mM sodium acetate; different pH), and 10 μg of GAD. The kinetics of these enzymes was determined under their optimal pH and temperature, and the concentrations of substrate (glutamate) ranged from 0.9 to 36 mM.

### Statistical analysis

All presented data in the bar charts and tables correspond to means ± standard deviation. Significant difference was carried out by using IBM SPSS Statistics 20.0 version.

## Results

### Genomic survey identifies common distribution of *gad* operon in *Lb. brevis*

**Figure 1:**
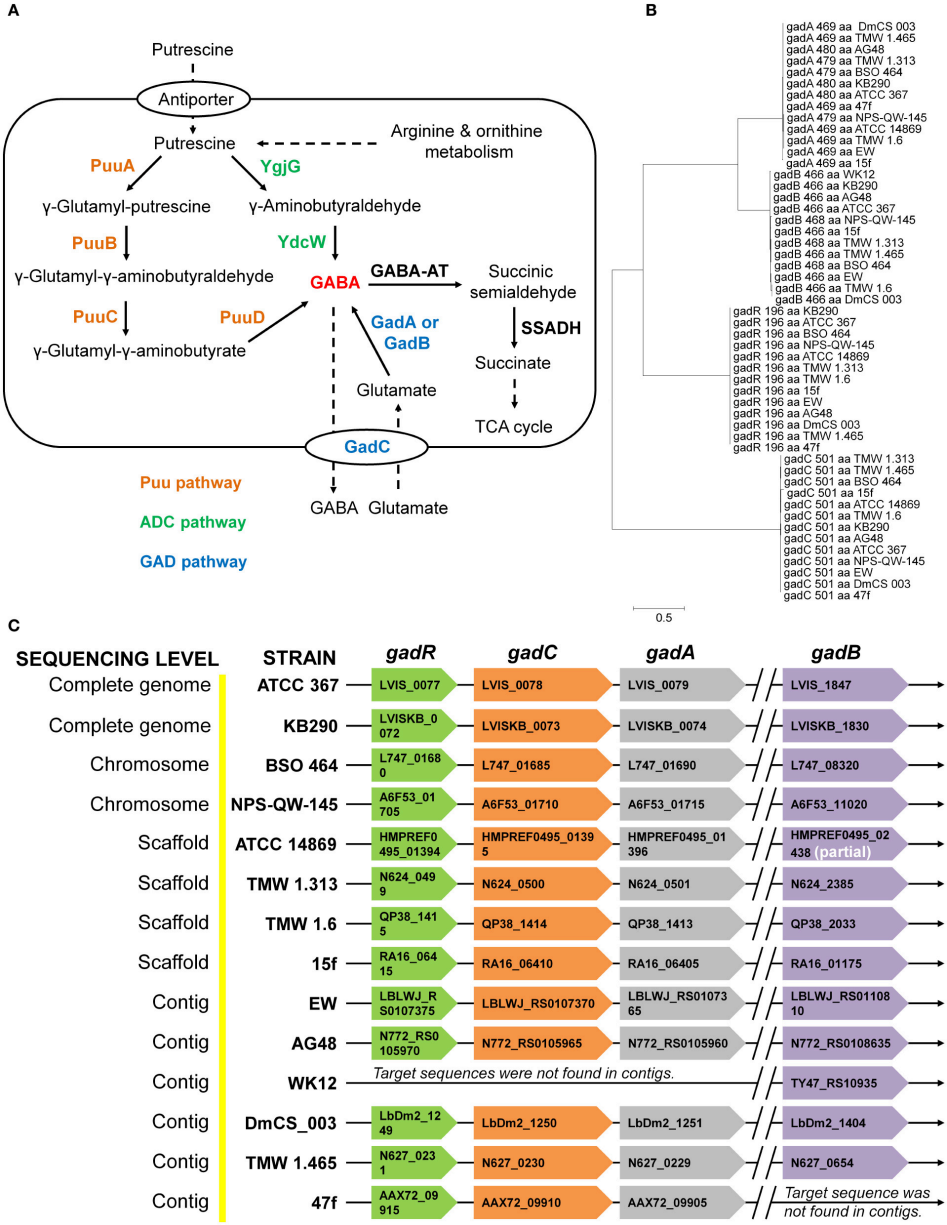
**Common distribution and arrangement of ***gad*** operon and genes encoding glutamic acid decarboxylase in the genomes of ***Lb. brevis*****. (A) General GABA production in bacteria from GAD pathway and putrescine degradation pathways (Puu pathway and ADC pathway). **(B)** Phylogeny of amino acids sequences of four components in *gad* operon demonstrating two isoforms of glutamate decarboxylases and the highly-conserved four genetic components in *Lb. brevis*. **(C)** Common distribution of *gad* operon in all the sequenced strains of *Lb. brevis*. Denotations: GABA-AT, GABA aminotransferase; SSADH, succinic semialdehyde dehydrogenase; *gadA*, glutamate decarboxylase isoform A; *gadB*, glutamate decarboxylase isoform B, *gadR*, transcriptional regulator; *gadC*, Glu/GABA antiporter. The phylogenetic tree was generated from MEGA (version 6.0) after MUSCLE alignment of amino acids sequences of each component in *gad* operon. The length of each component (locus tag indicated) in *gad* operon is indicated in the braces. The chromosome of a model strain *Lb. brevis* NPS-QW-145 was completely sequenced in this study and its NCBI accession no. is CP015398. All gene loci and genome data were collected from NCBI genome database (genome assembly and annotation report) on 10 January 2016.

Currently, there have been two major pathways reported for GABA production either through the degradation of putrescine (Puu and ADC pathways) or via the decarboxylation of glutamate (GAD pathway) in bacteria (Figure 1A). However, GABA production from putrescine degradation is not common in LAB and bifidobacteria due to the absence of Puu and ADC pathways. Interestingly, genes (*gadA* and *gadB*) encoding glutamate decarboxylases (GadA and GadB) and an intact *gad* operon including *gadR* (regulator), *gadA* and *gadC* (Glu/GABA antiporter) have been identified in all of the sequenced strains of *Lb. brevis* having varying origins excluding the one of strain WK12 with incomplete (contig levels) genome sequence (Figure 1C). The location of *gadB* is far away from *gad* operon in *Lb. brevis* suggesting there may be two different gene regulations for *gadA* and *gadB* in this species. However, arrangement of *gadA* and *gadC* close to each other in the genome could ensure the timely co-regulation of transcription and translation of *gadA* and *gadC* for GABA production (De Biase and Pennacchietti, 2012). Moreover, phylogenetic analysis of four gene components (*gadR, gadC, gadA*, and *gadB*) indicated the presence of two isoforms of glutamate decarboxylases, GadA and GadB, in *Lb. brevis*; all of the four components from varying *Lb. brevis* strains are highly conserved in this species (Figure 1B). This implies that co-existence of *gadA* and *gadB* in *Lb. brevis* contributes to its GABA synthesizing capacity.

### Global diversity of GABA-producing LAB and bifidobacteria

In order to gain a global view on the distribution of *gad* operon and genes encoding glutamate decarboxylases in LAB and bifidobacteria, a genomic survey was carried out on the sequenced LAB and bifidobacteria species, of which at least two strains have been sequenced and deposited in GenBank database (accessed on 10 January 2016). As shown in Table 2, many strains carry *gadA* or *gadB*, but *gadC* is only present in the genomes of limited strains indicating their strain-specific characteristic of GABA production. Among *gad* operon-positive strains, *Lb. brevis* is the only one species that has close to 100% probability to carry the intact *gad* operon in their chromosomes. All the sequenced strains of *B. dentium* possessed *gadBC* operon in their chromosomes. However, there is very little information on GABA production from *B. dentium* with only *gadB* gene, whereas there are several reports on high GABA production from *Lb. brevis* with two *gad* genes (Li and Cao, 2010; Dhakal et al., 2012; Wu and Shah, 2016). Thus, the present study focused on only *Lb. brevis* for its efficient GABA synthesis. Moreover, it was found that other common reported GABA-producing species such as *Lb. reuteri* and *Lc. lactis* showed strain-specific GABA biosynthesis at the genetic level.

**Table 2:** **Distribution of ***gad*** operon and genes encoding glutamate decarboxylases in the sequenced lactic acid bacteria and bifidobacteria**.

**Species**	**No. of sequenced strains**	**No. of strains with intact *gad* operon**	**No. of strains with genes encoding glutamate decarboxylase**
*Lb. brevis*	15	14 (**ATCC 367, KB 290**, ATCC 14869, TMW 1.6, 15f, DSM 20054, EW, AG48, DmCS_003, 47f, TMW 1.465, BSO 464, TMW 1.313, NPS-QW-145)	15 (**ATCC 367, KB 290**, ATCC 14869, TMW 1.6, 15f, DSM 20054, EW, AG48, WK12, DmCS_003, 47f, TMW 1.465, BSO 464, TMW 1.313, NPS-QW-145)
*Lb. plantarum*	50	0	49 (**WCFS1, JDM1, ST-III, ZJ316, P-8, 16, DOMLa, B21, 5-2, ZS2058, HFC8**, CMPG5300, CGMCC 1.557, ATCC 14917, UCMA 3037, FMNP01, NL42, PS128, L31-1, CGMCC 1. 2437, DSM 16365, DSM 13273, NC8, IPLA88, 2165, 2025, AY01, LP91, WJL, 4_3, 19L3, JCM1149, wikim18, AG30, Lp90, DmCS_001, 90sk, CIP104448, TIFN101, 8 RA-3, 38, SNU.Lp177, WJL, Nizo2877, WLPL04, 80, CRL 1506, SF2A35B, WHE 92)
*Lb. delbrueckii*	27	0	0
*Lb. helveticus*	19	0	0
*Lb. acidophilus*	15	0	0
*Lb. casei*	33	0	0
*Lb. paracasei*	52	0	0
*Lb. rhamnosus*	61	0	0
*Lb. reuteri*	18	6 (100-23, mlc3, lpuph, TMW1.656, TMW1.112, LTH5448)	7 (**TD1**, 100-23, mlc3, lpuph, TMW1.656, TMW1.112, LTH5448)
*Lb. iners*	16	0	0
*Lb. gasseri*	14	0	0
*Lb. fermentum*	19	0	5 (**F-6**, 28-3-CHN, NB-22, 39, 779_LFER)
*Lb. salivarius*	14	0	0
*Lb. johnsonii*	10	0	0
*Lb. sakei*	6	0	0
*Lb. buchneri*	4	2 (**NRRL B-30929**, DSM 20057)	2 (**NRRL B-30929**, DSM 20057)
*Lb. sanfranciscensis*	2	0	0
*Lb. kunkeei*	14	0	0
*Lb. pentosus*	3	0	0
*Lb. jensenii*	14	0	0
*Lb. ruminis*	9	0	0
*Lb. crispatus*	15	0	0
*Lb. amylovorus*	5	0	0
*Lb. paralimentarius*	5	0	0
*Lb. kefiranofaciens*	6	0	0
*Lb. oris*	3	3 (PB013-T2-3, F0423, DSM 4864)	3 (**J-1**, PB013-T2-3, F0423, DSM 4864)
*Lb. mucosae*	5	0	0
*Lb. florum*	3	0	0
*Lb. paraplantarum*	2	0	2 (**L-ZS9**, DSM 10667)
*Lb. curvatus*	3	0	0
*Lb. acidipiscis*	3	0	0
*Lb. fructivorans*	4	0	0
*Lb. coryniformis*	3	0	0
*Leu. mesenteroides*	12	0	0
*Leu. pseudomesenteroides*	4	0	0
*Leu. citreum*	7	0	0
*Leu. gelidum*	3	0	0
*Leu. lactis*	4	0	0
*P. acidilactici*	8	0	0
*P. pentosaceus*	6	0	0
*Lc. lactis*	66	29 (**Il1403, MG1363, KF147, NZ9000, CV56, KW2, NCDO 2118, KLDS 4.0325, AI06, S0**, Dephy 1, CNCM I-1631, YF11, TIFN2, LD61, CECT 4433, DPC6853, CRL264, K231, KF67, KF146, KF196, KF282, LMG9446, LMG9449, LMG14418, M20, ML8, UC317)	53 (**Il1403, MG1363, KF147, NZ9000, CV56, KW2, NCDO 2118, KLDS 4.0325, AI06, S0**, Dephy 1, CNCM I-1631, YF11, A12, TIFN2, TIFN4, LD61, 511, GE214, Bpl1, CECT 4433, 1AA59, A17, JCM 5805, Mast36, DPC6856, DPC6853, CRL264, ATCC 19435, E34, K231, KF7, KF24, K337, KF67, KF146, KF196, KF282, LMG8520, LMG8526, LMG9446, LMG9449, LMG14418, KF134, KF201, Li-1, M20, ML8, N42, UC317, NCDO895, GL2, LMG7760)
*Lc. garvieae*	17	5 (21881, 8831, TB25, Tac2, TRF1)	10 (21881, UNIUD074, 8831, TB25, LG9, IPLA 31405, Tac2, M14, Lg-ilsanpaik-gs201105, TRF1)
*S. thermophilus*	24	0	7 (**ND03**, TH1435, TH1477, 1F8CT, TH985, KLDS3.1012, CNCM I-1630)
*O. oeni*	62	0	0
*W. cibaria*	5	0	0
*B. longum*	55	0	0
*B. animalis*	28	0	0
*B. breve*	31	0	0
*B. bifidum*	23	0	0
*B. pseudolongum*	7	0	0
*B. dentium*	6	6 (**Bd1, JCM 1195**, ATCC 27679, JCVIHMP022, ATCC 27678, DSM 20436; *gadBC* only)	6 (**Bd1, JCM 1195**, ATCC 27679, JCVIHMP022, ATCC 27678, DSM 20436)
*B. adolescentis*	9	0	8 (**22L, BBMN23**, L2-32, DSM 20087, 150, 2789STDY5834850, 2789STDY5834852, IVS-1)
*B. thermacidophilum*	5	0	0
*B. thermophilum*	4	0	0
*B. pseudocatenulatum*	7	0	0
*B. kashiwanohense*	4	0	0
*B. asteroides*	5	0	0
*B. angulatum*	5	0	5 (**JCM 7096**, LMG 11039, DSM 20098_1, DSM 20098_2, GT 102)
*B. catenulatum*	4	0	0
*B. scardovii*	4	0	0
*B. gallicum*	3	0	0

*All the sequenced strains with all assembly levels (complete genome, chromosome, scaffold & contig) were included in this table; all the data was generated from NCBI genome database (genome assembly and annotation report) on 10 January 2016. Strain ID highlighted in bold indicates its level of complete genome assembly. Denotations: Lb., Lactobacillus; Leu., Leuconostoc; P., Pediococcus; Lc., Lactococcus; S., Streptococcus; O., Oenococcus; W., Weissella; B., Bifidobacterium*.

Although, there are several reports on GABA-producing *Lb. plantarum* without sequencing their genomes (Li and Cao, 2010; Wu and Shah, 2016), in this study we found that *gadC* is absent in all the sequenced strains of *Lb. plantarum* (Table 2). Hence, a fermentation study was carried out using the sequenced *Lb. plantarum* WCFS1 as a model organism. Although, there was an incomplete pathway for GABA metabolism in this organism based on KEGG pathway (Figures S1A,B), its viable counts, *gadB* transcripts level, acid profiles and GABA production did not significantly change after glutamate supplementation to the medium (Figures S1C–H). Although *gadB* transcript was increased in the stationary phase suggesting an enhanced GABA synthesis, *gadC* in LAB is required and specific for exchanging extracellular glutamate with intracellular GABA (Figure S1D). Our cultivation experiment on *Lb. plantarum* WCFS1 complied with previous gene knock-out studies in other bacteria with intact *gad* operon that GadC is an important element for GABA production (Cotter et al., 2001; Lu et al., 2013).

### Diverse and constant acid resistance systems in *Lb. brevis*

**Figure 2:**
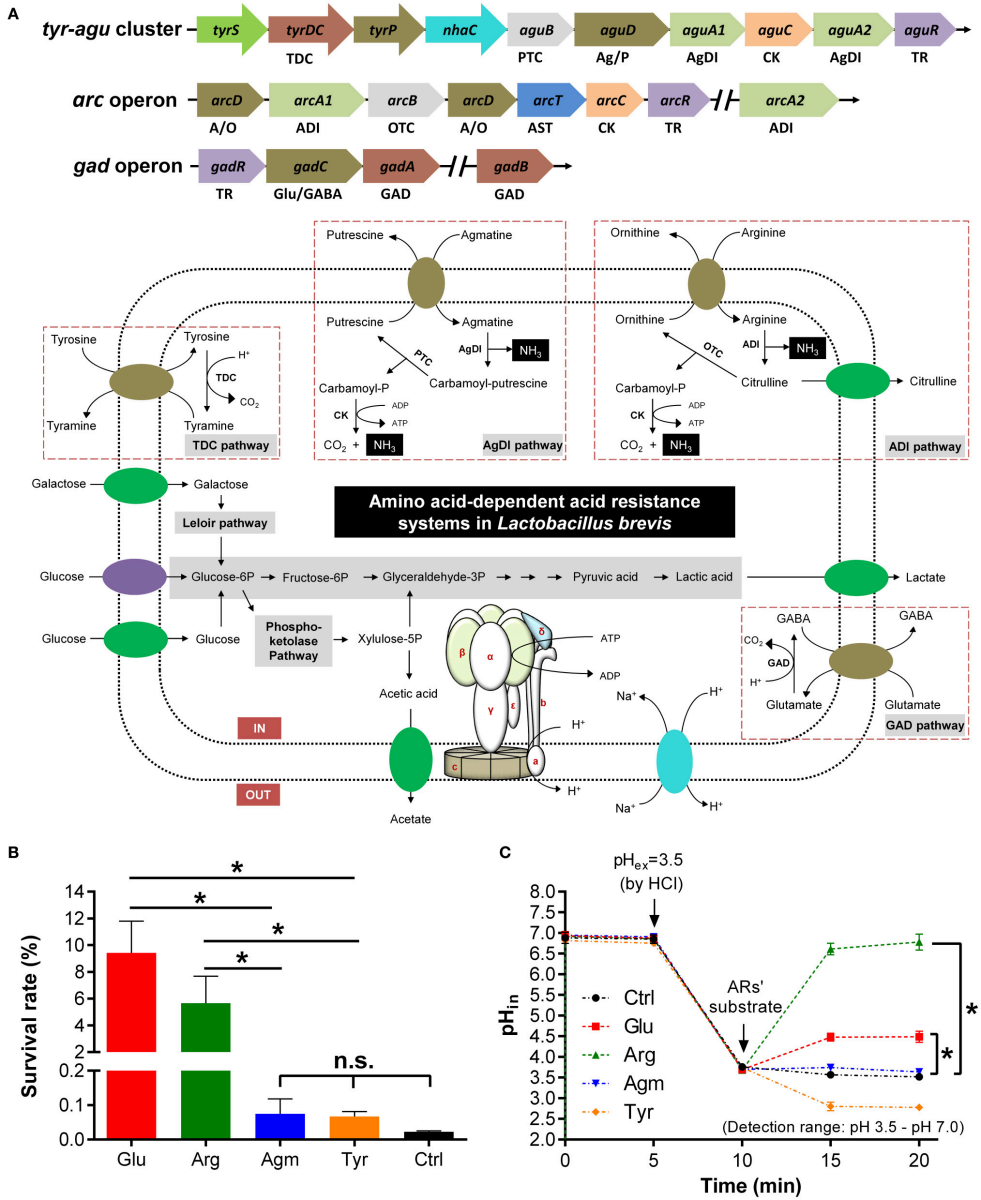
**GAD system in ***Lb. brevis*** improves cell viability by maintaining intracellular pH homeostasis. (A)** Carbohydrate metabolism and amino acid-dependent acid resistance (AR) systems in the model strains *Lb. brevis* 145. **(B)** Effect of amino acid-dependent ARs' substrates on survival rate of *Lb. brevis* cells (12-h cultures; acid-adapted cells) during acid resistance assay (37°C and 2-h incubation) carried out in Lactobacilli MRS medium (pH 2.5). **(C)** Effect of amino acid-dependent ARs' substrates on intracellular pH (pH_in_) of *Lb. brevis* cells (3-h cultures; non-acid-adapted cells) upon acid challenge tested at 37°C (extracellular pH–pH_ex_ decreased from pH 6.5 to pH 3.5. Glutamate, arginine and agmatine were dissolved in PBS buffer (pH 3.5) and tyrosine was dissolved in 0.1 M hydrochloric acid (HCl; after addition of tyrosine, pH_in_ of the cell was out of detection range but was still calculated from the equation of standard curve (pH_in_ = −0.1141 × ${\text{RFU}}_{488/435}^{2}$ + 1.4035 × RFU_488/435_ + 2.6307; *R*^2^ = 0.9849; pH range: 3.5–7.0) of pH and RFU_488/435_ (RFU, relative fluorescence units). Cells were suspended in phosphate-buffered saline but not citrate-based buffer for pH_in_ measurements ranging from pH 3.5 to pH 7.0. Denotations: GAD, glutamate decarboxylase; TDC, tyrosine decarboxylase; PTC, putrescine carbamoyltransferase; OTC, ornithine carbamoyltransferase; ADI, arginine deiminase; AgDI, agmatine deiminase; CK, carbamate kinase; TR, transcriptional regulator; A/O, arginine/ornithine antiporter; Ag/P, agmatine/putrescine antiporter; Glu/GABA, glutamate/GABA antiporter. Experiments were performed in triplicates and data is presented as mean ± standard derivation (SD). ^*^p < 0.05; n.s., not significant.

A genomic survey on the distribution of amino acid-dependent AR systems in all the sequenced strains of *Lb. brevis* was carried out. Except for universal amino acid-dependent AR systems including F_0_F_1_-ATPase system and amino acid/cation:proton antiporter, extra amino acid-dependent AR systems including GAD system, tyrosine decarboxylase (TDC) system, agmatine deiminase (AgDI) system and arginine deiminase (ADI) system are commonly present in all sequenced strains of *Lb. brevis* (Figure 2A and Table 3).

**Table 3:** **Acid resistance systems in the sequenced ***Lactobacillus brevis*** based on NCBI annotated protein database**.

**Strain**	**Sequencing level**	**F_0_F_1_-ATPase system**	**Cation:proton antiporter**	**GAD system**	**TDC system**	**ADI system**	**AgDI system**	**Urea system**
ATCC 367	Complete	√	√	√	√	√	√	×
KB290	Complete	√	√	√	√	√	√	×
NPS-QW-145	Chromosome	√	√	√	√	√	√	×
BSO 464	Chromosome	√	√	√	√	√	√	×
ATCC 14869	Scaffold	√	√	√	√	√	√	×
15f	Scaffold	√	√	√	√	√	√	×
TMW 1.313	Scaffold	√	√	√	√	√	√	×
TMW 1.6	Scaffold	√	√	√	√	√	√	×
EW	Contig	√	√	√	√	√	√	×
AG48	Contig	√	√	√	√	√	√	×
WK12	Contig	√	√	Incomplete	√	√	√	×
DmCS_003	Contig	√	√	√	√	√	√	×
TMW 1.465	Contig	√	√	√	√	√	√	×
47f	Contig	√	√	√	√	√	√	×

*All the data was generated from NCBI genome database (genome assembly and annotation report) on 10 January 2016. The incomplete GAD system in strain WK12 may be due to the incomplete sequencing level*.

### Activation of GAD system in *Lb. brevis* upon challenge with exogenous acid

**Figure 3:**
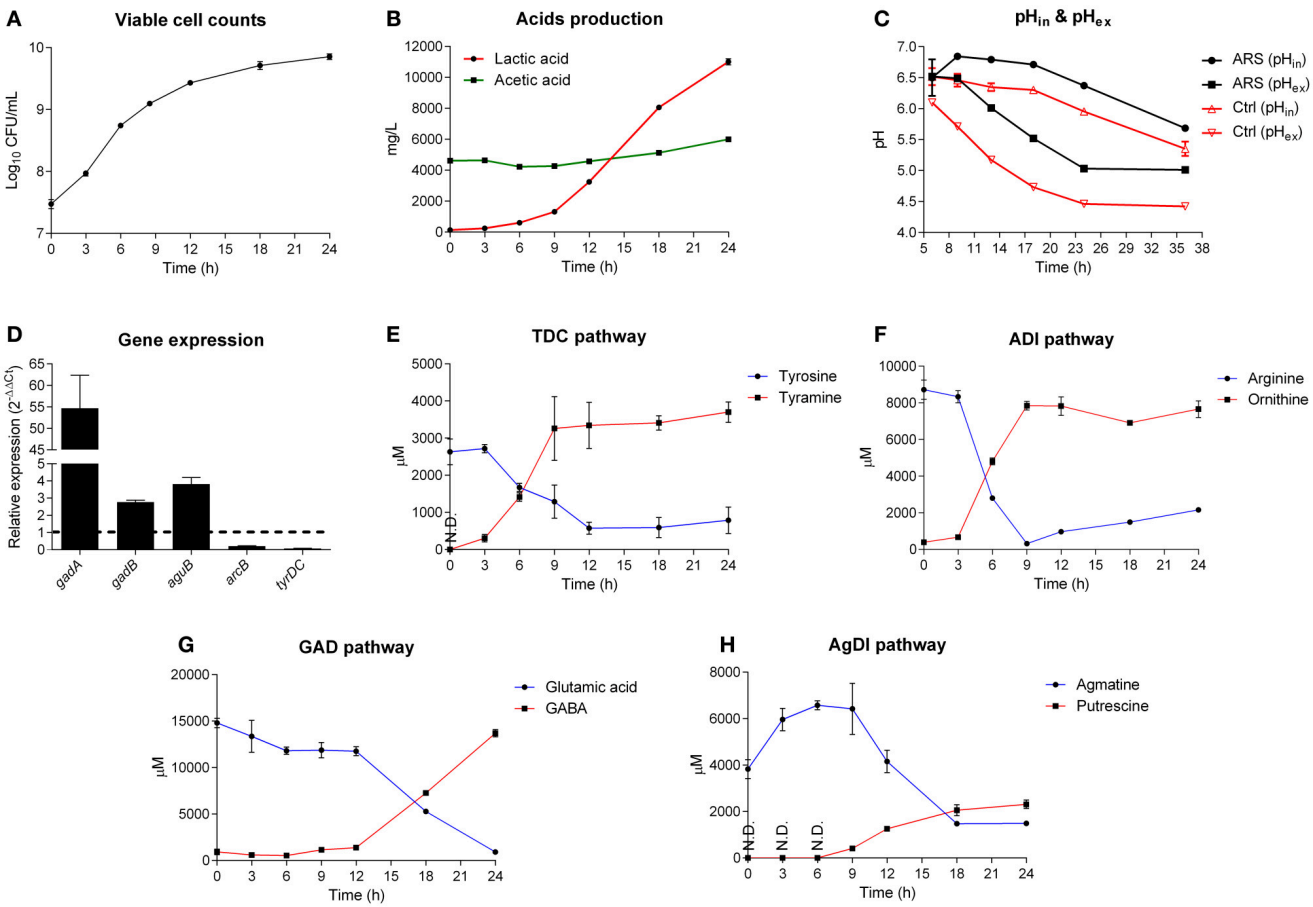
**GAD system is a major contributor for acid resistance of ***Lb. brevis***. (A)** Growth curve of *Lb. brevis* in lactobacilli MRS medium supplemented with extra glutamate, arginine, agmatine and tyrosine. **(B)** Lactic acid and acetic acid production. **(C)** Intracellular pH (pH_in_; cFDA-SE as the probe) and extracellular pH (pH_ex_) of *Lb. brevis* cells incubated in lactobacilli MRS medium with (ARS) or without (as control) amino acid-dependent ARs' substrates. **(D)** Relative gene expression of key genes of different amino acid-dependent AR systems in *Lb. brevis* at 18 h normalized to that at 6 h of cultivation. **(E–H)** Changes in the concentrations of the end products from each amino acid-dependent AR system. The experiment was carried out in triplicates and data was presented as mean ± standard derivation (SD).

Although, the contribution of AR systems for acid resistance in other LAB such as *Lb. reuteri* has been characterized (Teixeira et al., 2014), individual contribution of above AR systems to resist exogenous acids in *Lb. brevis* has not been well demonstrated. In this study, it was found that both glutamate and arginine were more effective in increasing the survivability of *Lb. brevis* significantly (*p* < 0.05) when exposed to acidic condition (pH 2.5; HCl-adjusted) for 2 h than that by agmatine and tyrosine (Figure 2B). Moreover, both glutamate and arginine were also able to increase the intracellular pH of *Lb. brevis* significantly (*p* < 0.05) than agmatine and tyrosine after challenging cells with hydrochloric acid (Figure 2C). This suggests that glutamate and arginine increased cell viability of *Lb. brevis* by increasing intracellular pH of the cell thus maintaining its metabolic activity. However, the increased level of intracellular pH of *Lb. brevis* cells by arginine and glutamate differed greatly. This may be due to the stages of cells selected for acid resistance experiment (12-h culture; acid-adapted cells) and acid challenge assay (3-h cultures; non-acid-adapted cells). The stages of cells may affect the contents of key cytosolic enzymes including GadA, GadB, ADI, and OTC from the four amino acid-dependent AR systems (Figure 3D). In addition, the increase in the intracellular pH by glutamate may be explained by GABA synthesis that consumed cytosolic protons in *Lb. brevis* upon acid challenge. Since the function of GadC is activated under acidic conditions (Ma et al., 2012), GAD system in *Lb. brevis* may be activated by acid challenge.

### GAD system in *Lb. brevis* is a major contributor for resisting endogenous acid

To understand the association between GAD system for endogenous acid (lactic acid) resistance and GABA production capacity of *Lb. brevis*, a 24-h course of cultivation experiment on *Lb. brevis* 145 in Lactobacilli MRS broth supplemented with four amino acid-dependent AR's substrates (glutamate, arginine, agmatine and tyrosine) was carried out. Firstly, it was observed that the AR's substrates were able to increase the intracellular pH of *Lb. brevis* cells by about 0.5 compared to that in *Lb. brevis* without the AR's substrates supplementation (Figures 3A–C). In addition, results indicate that *Lb. brevis* maintained its intracellular pH level in the near-neutral range (pH 5.5–6.5) or close to neutral range (pH 6.5–7.0) when extracellular pH decreased spontaneously by secreting protons during its lactic acid production (Figures 3B,C). Moreover, gene transcription of *gadB* and *aguB* increased by at least 2 fold whereas *gadA* mRNA was highly up-regulated by about 55 fold at the point of 18 h (stationary phase) when compared to that at 6 h (logarithmic phase) (Figure 3D). In combination with the higher rate of GABA production at 18 h than that at 6 h (Figure 3G), it appears that GAD system was more active in stationary phase than that in lag and logarithmic phases. In general, changes in the end metabolites of four amino acid-dependent AR systems also demonstrated that utilization of the AR's substrates varied with phases. Based on the metabolic data in Figures 3E–H and gene expression data in Figure 3D, TDC and ADI pathways were active in early cultivation (Figures 3E,F) but were down-regulated in late cultivation (Figure 3D), while GAD and AgDI pathways were more active in stationary phase (Figures 3G,H). Although a gene encoding lysine decarboxylase was found in *Lb. brevis* 145, the lysine content little changed suggesting an inactive state of this AR system (Figure S2).

The acid resistance and challenge assays may not be able to indicate actual contributions of AgDI and TDC pathways for resisting acids (Figures 2B,C); however, changes in the content of metabolites during different cultivation stages evidenced that all the four amino acid-dependent AR systems contributed to intracellular pH homeostasis (Figure 3C). Since GABA production from *Lb. brevis* was increased in the stationary and late stages, its GAD system played an important role in bacterial acid resistance because its lactic acid accumulated largely during late cultivation (Figure 3B).

### GadA supports GABA synthesis in *Lb. brevis* toward a weak pH range

**Figure 4:**
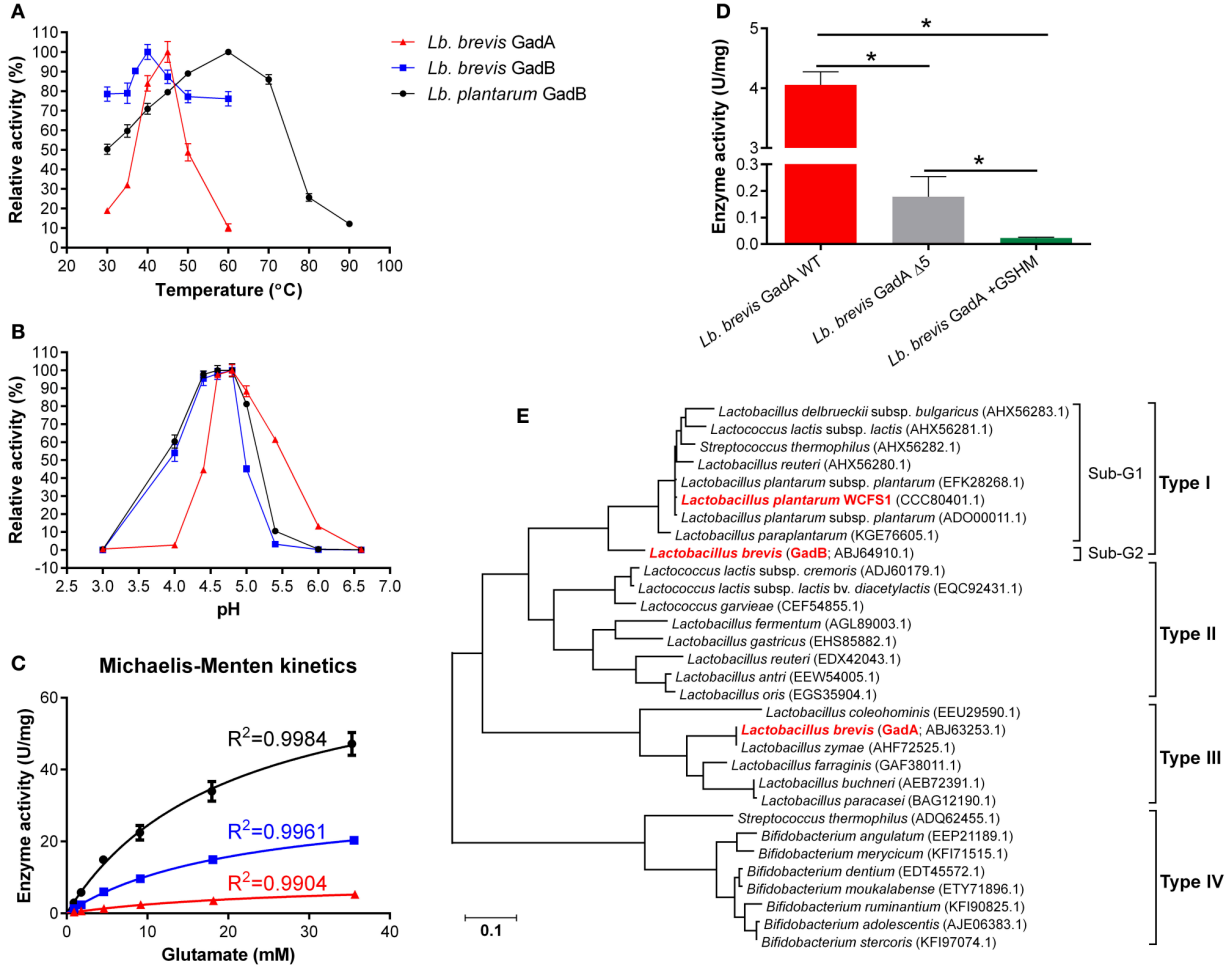
**GadA supports GABA synthesis in ***Lb. brevis*** toward a weak pH range of cytosolic acidity. (A)** Temperature effect on Gads tested under constant pH 4.8. **(B)** Acidity effect on Gads tested under constant 37°C. **(C)** Enzyme kinetics tested under their optimal pH and temperature. **(D)** Activities of wild-type *Lb. brevis* GadA and its mutants. **(E)** Phylogeny of representative Gads from LAB and *Bifidobacterium*. The well-characterized *Lb. plantarum* GadB was used as a reference Gad. The phylogenetic tree was generated from MEGA (version 6.0) after MUSCLE alignment of amino acids sequences of Gads. GraphPad Prism version 6.0 was used to generate kinetic curves of three Gads. The enzyme assay was carried out in duplicates and data is presented as mean ± standard derivation (SD). ^*^p < 0.05.

Two Gads, GadA and GadB, from *Lb. brevis* and a reference type I GadB from *Lb. plantarum* were hetero-expressed and purified for enzyme assay (Figure S3). Two mutants of *Lb. brevis* GadA (wild-type; WT), *Lb. brevis* GadA +GSHM (extra GSHM to N-terminus) and *Lb. brevis* GadA Δ5 (5 amino acids deleted from N-terminus), were constructed in this study. As shown in Figure 4A, *Lb. brevis* GadA (WT) exhibited a narrow activity spectrum toward the change of temperature, but still retained about 50% of the highest activity at 37°C, whereas *Lb. brevis* GadB and *Lb. plantarum* GadB were heat-stable enzymes. More interestingly, *Lb. brevis* GadB and *Lb. plantarum* GadB retained similar catalytic spectrum in the range of pH 3.0–5.5 while *Lb. brevis* GadA (WT) exhibited activity toward a weak acidity range (pH 5.5–6.6) (Figure 4B). The *in vitro* enzyme kinetic assay (Figure 4C and Table 4) demonstrated that catalytic efficiency of *Lb. brevis* GadA (WT) was largely lower than that of two GadB enzymes. However, these three Gads had a very similar K_m_ value suggesting a similar level of substrate saturation (Table 4). Moreover, *Lb. brevis* GadA (WT) exhibited a significant (*p* < 0.05) activity than its two mutants with modifications to its N-terminus (Figure 4D). Previous gel filtration studies have indicated the tetramer state of *Lb. brevis* GadA (Hiraga et al., 2008), the monomer state of *Lb. brevis* GadB (Yu et al., 2012; Shi et al., 2014) while *Lb. plantarum* GadB function as dimers (Shin et al., 2014). This suggests that N-terminus of *Lb. brevis* GadA may be critical to the tetramer formation via incorporation to its N-terminus. The phylogenetic analysis of representative Gads from LAB and *Bifidobacterium* highlighted that *Lb. brevis* GadA was a type III group of Gads which differed largely from *Lb. brevis* GadB (sub-group 2) and *Lb. plantarum* GadB (sub-group 1) which were classified in type I Gads (Figure 4E). Overall, *Lb. brevis*, as a cell factory, exhibited GABA biosynthesizing capacity in a board range of acidity, especially in near-neutral pH range (pH 5.5–6.5).

**Table 4:** **Kinetic parameters of ***Lb. brevis*** GadA, ***Lb. brevis*** GadB and ***Lb. plantarum*** GadB under optimal conditions**.

**Kinetic parameter**	***Lb. brevis* GadA (pH 4.8, 45°C)**	***Lb. brevis* GadB (pH 4.8, 40°C)**	***Lb. plantarum* GadB (pH 4.8, 60°C)**
V_max_ (μmol·min^−1^·mg^−1^)	9.157 ± 0.4542	32.56 ± 0.8842	73.33 ± 4.671
K_m_ (mM)	26.95 ± 2.437	21.39 ± 1.142	20.02 ± 2.557
k_cat_ (min^−1^)	490.18 ± 24.31	1757.91 ± 47.74	3954.76 ± 251.91
k_cat_/K_m_ (mM^−1^·min^−1^)	18.19	82.18	197.54

*Kinetic parameters were based on Michaelis-Menten equations (Figure 4C) generated from GraphPad Prism version 6.0*.

## Discussion

GABA synthesis can also be derived from putrescine degradation process via either Puu pathway or arginine decarboxylase (ADC) pathway as shown in Figure 1A in some bacterial species such as *Escherichia coli* (Kurihara et al., 2008, 2010); however, based on our KEGG survey on arginine and proline metabolism map of complete sequenced bacterial strains, these two pathways are not commonly present in LAB and bifidobacteria (data not shown). Thus, GABA production from LAB and bifidobacteria is mainly derived from GAD pathway if it is present in them. Due to the important biofunctionalities of GABA and GABA-producing LAB and bifidobacteria, several studies have characterized GABA production from individual strains (Li and Cao, 2010; Dhakal et al., 2012; Diana et al., 2014; Wu and Shah, 2016). The diversity of GABA-producing LAB and bifidobacteria has been poorly understood either at genetic level or from experimental insights. Our genomic survey conducted on most of the sequenced strains of LAB and bifidobacteria released in GenBank has clearly indicated that the genetics of *Lb. brevis* differed with other species of LAB and bifidobacteria in terms of GABA production: (1) an intact *gad* operon, and (2) two genes (*gadA* & *gadB*) encoding glutamate decarboxylases in their genomes (Figure 1C and Table 2). Thus, GABA production from *Lb. brevis* appears to be species-specific based on our current genetic observation (Table 2) and previously reported GABA yields from various strains of *Lb. brevis* (Li and Cao, 2010; Dhakal et al., 2012; Wu and Shah, 2016). This differs with the strain-specificity of GABA biosynthesis in other species such as *Lactococcus lactis* and *Lb. reuteri* (Table 2). To our knowledge, this is the first global insights into genetic diversity of GABA producer among above food-grade bacteria.

It is clear that GAD system is one of AR systems in bacteria to cope against acidic environments thus allowing them to maintain metabolic potential and cell viability (De Biase and Pennacchietti, 2012). Multiple AR systems including universal and amino acids-based AR systems function in the bacteria, but the contribution of GAD system to bacterial acid resistance has not been clearly demonstrated so far. Since *Lactobacillus* genus including *Lb. brevis* is a high lactic acid producer (Leroy and De Vuyst, 2004), excessive intracellular protons from endogenous or exogenous acids can be neutralized or eliminated by their AR systems. Among the four amino acid-dependent AR systems that were commonly identified in *Lb. brevis*, our results indicate the major contribution of GAD system for acid resistance and challenge in this organism upon either short-term or long-term acidic exposure, and either extracellular or intracellular acidification (Figures 2, 3). For instance, high amount of lactic acid was produced and accumulated in the medium during stationary phase of *Lb. brevis* cultivation, this organism utilized the highly active GAD system that decarboxylated glutamate into GABA greatly at this stage contributing to the increase in cytosolic pH (Figure 3). This highlights that *Lb. brevis*, a lactic acid producer, applies GAD system as the main strategy for its acid resistance thus reflecting its high capacity of GABA production.

Considering high GABA production from *Lb. brevis* and its GAD system, it is crucial to understand the mechanism of efficient GABA synthesis for this phenomenon. Several studies have reported individual *Lb. brevis* GadB tested either in acetate buffer or citrate-phosphate buffer exhibiting its activity spectrum toward acidic conditions (pH 3.5–5.5) but not near-neutral pH (pH 5.5–6.5; Yu et al., 2012; Seo et al., 2013; Lin et al., 2014), but the activity of only one GadA from *Lb. brevis* IFO 12005 has been reported twice focusing on its activation of enzymatic activity during *in vitro* conditions (Ueno et al., 1997; Hiraga et al., 2008). However, these studies did not provide systematic and comparative insights into this decarboxylation machinery in *Lb. brevis*. In general, *Lb. brevis* GadA retained its activity from pH 4.0 to pH 6.6 in the sulfate buffer (Figure 4B). This showed similar activity spectrum, particularly in near-neutral pH range (pH 5.5–6.5), with another reported *Lb. brevis* IFO 12005 GadA tested in pyridine-HCl buffer after sulfate activation and enzyme purification via gel filtration (Ueno et al., 1997; Hiraga et al., 2008). The characteristic of *Lb. brevis* GadA differs greatly with *Lb. brevis* GadB since the former requires enzyme activation by high concentrations of sulfate (Hiraga et al., 2008). As for *Lb. brevis* GadB, our study and previous reports showed its active activity in a more acidic range (pH 3.0–5.5; Figure 4B; Yu et al., 2012; Seo et al., 2013). Taken together, GadA and GadB in *Lb. brevis* cells may help them synthesize GABA toward a broad range of intracellular pH (pH 3.0–6.6).

As shown in Figure 3C, intracellular pH of *Lb. brevis* cells during a standard 24-h course of cultivation in MRS medium was in the range of pH 5.8–6.5. This indicates that glutamate decarboxylation may be very slow due to limited activities of both Gads in this near-neutral pH range (pH 5.5–6.5). Moreover, pH_in_ of *Lb. brevis* cells decreased in the late incubation (24–36 h) thus may increase GABA synthesis by the enhanced activities of both GadA and GadB. This may be able to explain why resting cells could be used for GABA production (Zhang et al., 2012). Moreover, transmembrane potential, which contributes to amino acid-dependent acid resistance in bacteria, could be increased by glutamate decarboxylation (Teixeira et al., 2014). The transmembrane ΔpH in the ARS group was less than that in the control after the incubation of 9 h (Figure 3C) indicating that transmembrane potential in ARS group may be higher than that in the control; this effect may be attributed by glutamate decarboxylation in *Lb. brevis*.

The tetramer formation of *Lb. brevis* GadA *in vitro* needs high content of sulfate ions which is not similar to that in the *in vivo* conditions (Hiraga et al., 2008). This may be due to the fact that *in vitro* catalytic activity does not fully represent *in vivo* situation of the enzyme, though in general it concurs to that *in vivo* in bacteria (Davidi et al., 2016). However, we observed that glutamate was almost decarboxylated into GABA within 24 h (Figure 3G), even at the supplementation level of 10 g/L of monosodium glutamate (Wu and Shah, 2015), and GadA showed similar spectrum of enzyme activity either in sulfate buffer (Figure 4D) or in pyridine-HCl buffer (Hiraga et al., 2008). This may suggest that the pH value rather than the composition of the buffer affects the activity of GadA greatly after the activation process for GadA.

The near-neutral intracellular pH of *Lb. brevis* cells limited activities of cytosolic Gads though GadA and GadB were highly regulated (Figures 3C,D, 4B). Thus, approaches to improve their activities in near-neutral pH (pH 5.5–6.5) would be of economic and biological importance. Two studies that applied mutation strategy to *Lb. brevis* GadB demonstrated that these mutants extended their activities toward near-neutral acidity (Yu et al., 2012; Shi et al., 2014). However, we observed the extended activity of GadA in near-neutral pH range (pH 5.5–6.5) supporting GABA synthesis in *Lb. brevis* offering a novel insight into the diversity of Gads from LAB and bifidobacteria (Figure 4B). Although detailed regulatory mechanism for GABA synthesis in *Lb. brevis* is not clear, the *gadA* mRNA transcript became more abundant than *gadB* mRNA transcript in *Lb. brevis* at the stationary phase; this may contribute to its high GABA production by this organism during late cultivation (Figures 3D,G). The classification of Gads from LAB and bifidobacteria also indicated that *Lb. brevis* GadA and GadB were unique Gads differing with other Gads (Figure 4E). In general, we concluded that *Lb. brevis* GadA, a novel type III Gad that may play a more important role than *Lb. brevis* GadB, exhibited its activity toward weak pH range (pH 5.5–7.0) of bacterial cytosolic acidity. This supports the efficient GABA synthesis in *Lb. brevis* as a microbial cell factory.

Apart from the contribution of GAD system to acid resistance during late cultivation of *Lb. brevis*, our results showed that its ADI system was active in early cultivation, specifically in lag and log phases (Figures 2, 3D,F). This implies that ADI pathway plays a key role in early acid resistance by alkali production.

In conclusion, GAD system was commonly distributed in *Lb. brevis* among various species of LAB and bifidobacteria, and was the major contributor for acid resistance thus exhibiting a high capacity of GABA production of the cell. GadA in GAD system played a key role in GABA synthesis via its extended activity toward a near-neutral pH range (pH 5.5–6.5) of cytosolic acidity of *Lb. brevis* cells. Further understanding on tetramer formation and structural insights of *Lb. brevis* GadA would be necessary to unravel the decarboxylation machinery in a weak acidic condition.

## Author contributions

QW and NS designed the study; HT and EK performed the draft genome sequencing of *Lactobacillus brevis* NPS-QW-145; QW performed the experiments; QW and YL analyzed the data; QW and NS wrote the manuscript.

## Funding

This work is partially supported by Seed Funding Programme for Basic Research (project code: 201511159156), University Research Committee, The University of Hong Kong.

### Conflict of interest statement

The authors declare that the research was conducted in the absence of any commercial or financial relationships that could be construed as a potential conflict of interest.
