# Supplementary material for: Common Distribution of gad Operon in Lactobacillus brevis and its GadA Contributes to Efficient GABA Synthesis toward Cytosolic Near-Neutral pH
Source: Front Microbiol. 2017 Feb 14;8:206. doi: 10.3389/fmicb.2017.00206 (PMC5306213; doi:10.3389/fmicb.2017.00206)
Supplement: Supplementary file 1 [file Presentation1.PDF]

# Supplementary material

**This SM file includes:**

Supplementary materials and methods

Supplementary references

Supplementary Figures from S1 to S3

## Supplementary materials and methods

*Genome sequencing, assembly and annotation*

The genomic DNA from *Lb. brevis* 145 was extracted using the ZR Fungal/Bacterial DNA MidiPrep™ Kit (Zymo Research, Irvine, CA, USA) according to the manufacturer's instructions. About 50 ng of genomic DNA was subjected to construct shotgun metagenomic library using Nextera® DNA Library Preparation kit (Illumina, San Diego, CA, USA) according to the manufacturer's protocol. Briefly, gDNA was simultaneously fragmented by Nextera transposome, followed by a clean-up step for tagmented gDNA. The purified gDNA fragments were then amplified using via a limited-cycle PCR program (5 cycles) that appended sequencing primers (Rd1 SP and Rd2 SP), sample indexes (index 1 and index 2), and sequencing adapters (P5 and P7) to each gDNA fragment. The tagged gDNA fragments were subsequently purified using AMPure XP beads (Beckman Coulter Genomics, Danvers, MA, USA) to remove short fragments. The qualified libraries were then quantified via Qubit assay (Fisher Scientific, Ottawa, ON, Canada), and its quality including the size distribution was later checked by Aglient 2100 Bioanalyzer (Aglient Technologies, Santa Clara, CA, USA). Then, libraries were pooled and subjected for 300 bp paired-end sequencing on a Miseq platform (Illumina, San Diego, CA, USA). Services of paired-end reads (5-6 kb) library construction and HiSeq4000 sequencing for *Lb. brevis* 145 were provided by BGI Tech Solutions (Hong Kong) Co., Limited.

*De novo* assembly of paired-reads (~300 bp) generated form MiSeq platform was carried out using command line-based Newbler (version 2.8) resulting in 15 contigs; another paired-end reads (6 kb-span) was used to scaffold above contigs by SSPACE (Hunt et al., 2014); gaps in scaffolds were extended by paired-end reads (6 kb-span) using GapFiller (Boetzer and Pirovano,

2012). Mapping all the scaffolds to the reference genome of *Lb. brevis* KB290 by CONTIGuitor (Galardini et al., 2011) generated a draft genome of chromosome with gaps. Sequences of those gaps remained in the draft genome were obtained by general PCR and Sanger sequencing method. Additionally, a set of primers were designed based on two ends of the draft genome; PCR amplification and Sanger sequencing were applied again to confirm the circular status of the chromosome achieving the complete genome sequence of the chromosome of *Lb. brevis* 145. Complete chromosome sequence was further annotated by using NCBI Prokaryotic Genome Annotation Pipeline.

*Cloning, hetero-expression and purification of glutamic acid decarboxylases*

The full length of two genes – *gadA* and *gadB* from *Lb. brevis* 145 and *gadB* from *Lb. plantarum* WCFS1 were amplified using the primers listed in Table 1 resulting in the incorporation of restriction sites for *HindIII*, *NdeI*, *AgeI* and *EcoRI* enzymes (New England BioLabs, Ipswich, Massachusetts, USA) to both ends. Based on the availabilities of restriction sites in the normal expression vector – pET-28a(+), *NdeI* site, which is not present in the inserted Gad genes, was chosen for the expression of *Lb. plantarum* GadB (Shin et al., 2014), *Lb. brevis* GadB (Shi et al., 2014) and *Lb. brevis* GadA; another reason for choosing *NdeI* site was that less residues (GSHM) was retained to the N-terminus of the expressed Gad after the cleavage of his-tag by thrombin. The stop codon was remained in the Gad genes followed by another available restriction site in pET-28a(+). If the GAD enzyme requires intact N-terminus, another expression vector – pRSETA-SUMO, an in-house expression plasmid based on pRSET-A (Invitrogen) with an N-terminal His-SUMO tag, was used for Gad gene expression (Chan et al., 2012). Detailed information of plasmids, restriction sites selection, and primers was indicated in Table 1. The

PCR reaction volume included 0.2 µL of AccuPrime™ *Taq* High Fidelity (Invitrogen), 5 µL of 10X AccuPrime™ PCR Buffer II, 1 µL of each primer (10 µM), 5 µL of DNA template and add DNase-free water to a total volume of 50 µL. The amplification was carried out in a GeneAmp® PCR system 2700 (Applied Biosystems) with 30 cycles (94℃ for 20 s, 58℃ for 30 s, and 68℃ for 2 min). Then, PCR products were firstly checked by agarose gel electrophoresis and were then directly purified according to the manufacture's instruction of S.N.A.P.™ Gel Purification Kit (Invitrogen). The plasmid pET-28a(+) in *E.coli* DH5α and pRSETA-SUMO (in house expression vector containing both 6×his-tag and SUMO-tag) in *E. coli* XL1-Blue were isolated using alkaline lysis method. Both plasmids and PCR products were further digested by HindIII, NdeI, AgeI and EcoRI as per the manufacturer's instructions. After digestion, purification using S.N.A.P.™ Gel Purification Kit (Invitrogen) was performed again for both vectors and PCR products, which were ligated by T4 DNA ligase (New England BioLabs) at 16℃ for overnight resulting in the formation of pET-28a(+)-Lb-gadA, pET-28a(+)-Lb-gadB, pET-28a(+)-Lp-gadB, pRSETA-SUMO-Lb-gadA, pRSETA-SUMO-Lb-gadA Δ5. Above ligated plasmids were transformed into the competent cells of *E. coli* strains BL21(DE3) or BL21(DE3)pLysS. After 18-h activation in the LB plates (50 µg/L of kanamycin or ampicillin), colonies were selected and double-checked by colony-PCR amplification using T7 promoter/terminator primers. Colonies with positive inserts were cultured and sent to BGI Tech Solutions (Hong Kong) Co., Ltd. for Sanger sequencing.

Expression host cells of *E. coli* carrying positive inserts were cultured in LB broth containing 50 µg/L of kanamycin or ampicillin at 37 ℃. Isopropyl β-D-1-thiogalactopyranoside (IPTG; Sigma) was added at mid-exponential phase ($OD_{600}$ ~ 0.6) resulting in the final concentration of 0.5 mM in the medium. Cells were kept growing for another 12 h at 20℃, and were harvested by

centrifugation at 10,000 ×g and 4℃ for 20 min. Cells were washed again with ice-cooled saline buffer and were suspended in the ice-cooled binding buffer (20 mM sodium phosphate, 0.5 M sodium chloride, 20 mM imidazole, pH 7.4). Sonication was applied to disrupt the cells and hetero-expressed proteins released into the supernatant were passed through 0.22 μm membrane filter and were further purified via immobilized metal ion affinity chromatography (IMAC) by using 1-mL HisTrap™ HP column (GE Healthcare, Little Chalfont, UK) as per the manufacturer's instructions. Hetero-expressed proteins eluted in the buffer (20 mM sodium phosphate, 0.5 M sodium chloride, 500 mM imidazole, pH 7.4) were dialyzed against the dialysis buffer (300 mM sodium chloride, 5% (v/v) glycerol, 30 mM Tris, pH 7.5) at 4℃. Removal of his-tag and SUMO-tag from the N-terminus of proteins were achieved by adding thrombin or SUMO protease (Sigma) to the dialyzed protein samples and incubated at 4℃ for 2-3 hours. Target proteins after proteolytic cleavage were purified by Gel Filtration Chromatography equipped with using a HiLoad 16/60 Superdex 75 column (GE Healthcare). Fractions were collected and checked by sodium dodecyl sulfate–polyacrylamide gel electrophoresis (SDS-PAGE). Fractions with the same target band were pooled together and concentrated by Amicon® Ultra-15 Centrifugal Filters (Millipore). The concentration of proteins was determined by Bradford method using bovine serum albumin (BSA) as standard. The target protein bands excised from the Coomassie Brilliant Blue-stained SDS-PAGE gels were sent to HKU Center for Genomic Sciences for mass spectroscopy of protein identification service.

## Supplementary references


Boetzer, M., and Pirovano, W. (2012). Toward almost closed genomes with GapFiller. *Genome Biol.* 13(6):R56. doi: 10.1186/gb-2012-13-6-r56.

Chan, K.H., Lee, K.M., and Wong, K.B. (2012). Interaction between hydrogenase maturation factors HypA and HypB is required for [NiFe]-hydrogenase maturation. *PLoS One* 7(2):e32592. doi: 10.1371/journal.pone.0032592.

Galardini, M., Biondi, E.G., Bazzicalupo, M., and Mengoni, A. (2011). CONTIGuator: a bacterial genomes finishing tool for structural insights on draft genomes. *Source Code Biol. Med.* 6:11. doi: 10.1186/1751-0473-6-11.

Hunt, M., Newbold, C., Berriman, M., and Otto, T.D. (2014). A comprehensive evaluation of assembly scaffolding tools. *Genome Biol*. 15(3):R42. doi: 10.1186/gb-2014-15-3-r42.

Shi, F., Xie, Y.L., Jiang, J.J., Wang, N.N., Li, Y.F., and Wang, X.Y. (2014). Directed evolution and mutagenesis of glutamate decarboxylase from *Lactobacillus brevis* Lb85 to broaden the range of its activity toward a near-neutral pH. *Enzyme Microb. Tech.* 61-62**,** 35-43.

Shin, S.M., Kim, H., Joo, Y., Lee, S.J., Lee, Y.J., Lee, S.J., and Lee, D.W. (2014). Characterization of glutamate decarboxylase from *Lactobacillus plantarum* and its C-terminal function for the pH dependence of activity. *J. Agr. Food Chem.* 62**,** 12186-12193.

## Supplementary Figures

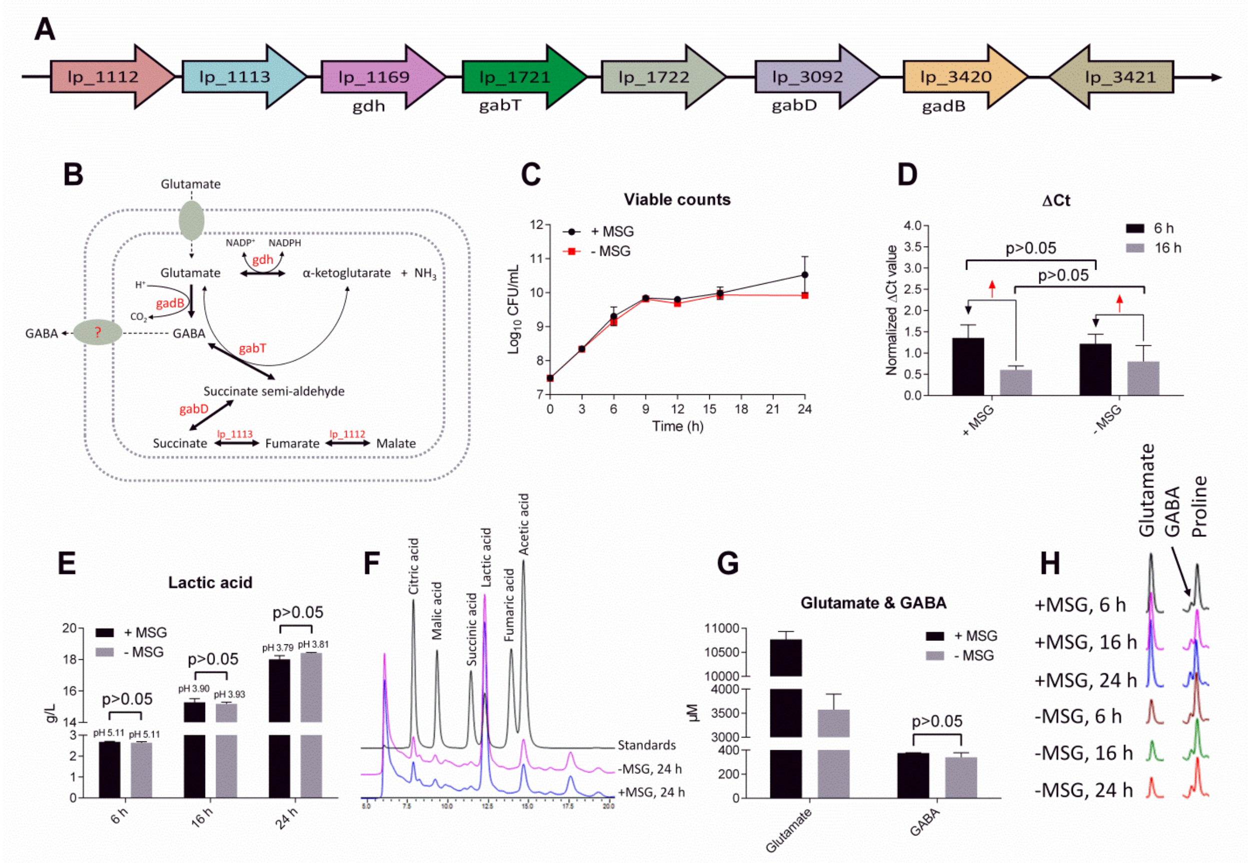


**Fig. S1. A GABA-producing *Lactobacillus plantarum* WCFS1 with *gadB* but lacking *gadC* in its genome demonstrated the important role of Glu/GABA antiporter for GABA production.** (**A**) Gene *gadC* was not identified in the genome of *Lb. plantarum* WCFS1. (**B**) KEGG metabolic pathway for GABA contains genes that are related to glutamate metabolism in *Lb. plantarum* WCFS1. (**C**) Viable cells of *Lb. plantarum* WCFS1 cultured in lactobacilli MRS medium supplemented with or without 1 g/L MSG. (**D**) Gene expression of *gadB*. The red upward arrow (↑) indicates the up-regulation (1< fold change < 2) of *gadB* at 16 h after normalization to ΔCt value at 6 h. (**E**) Lactic acid production. (**F**) HPLC spectra of acids profiles. (**G**) Residual glutamate and GABA content in the medium after 24-h incubation. (**H**) Changes in the content of glutamate and GABA in the 24-h course of incubation. The experiment was carried out in triplicates and data was presented as mean ± standard derivation (SD).

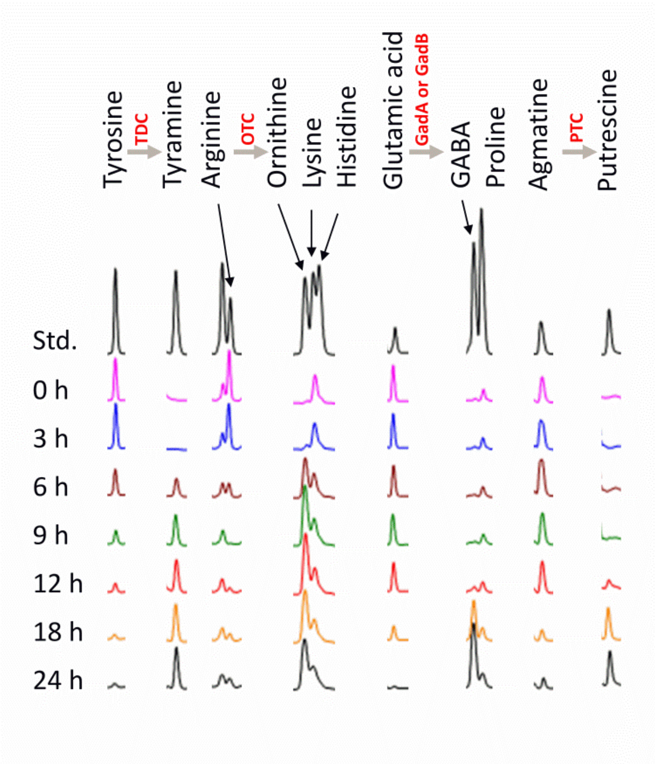


**Fig. S2. Changes in the peak area of HPLC chronograms for targeted amino acids and amines from amino acid-dependent acid resistance system in *Lb. brevis* 145.**

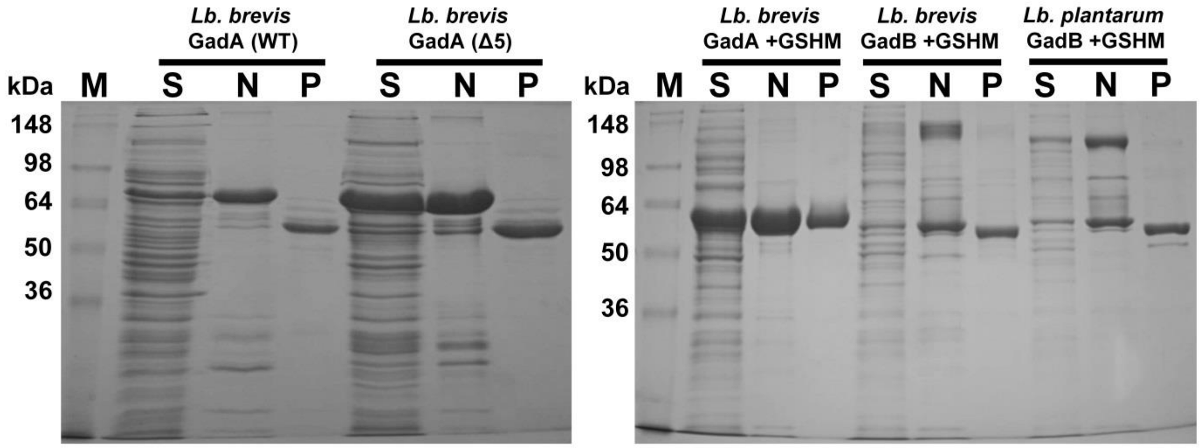


**Fig. S3. Expression, purification and identification of glutamate decarboxylases from *Lb. brevis* 145 and *Lb. plantarum* WCFS1.** For the expression and purification of *Lb. brevis* GadA (WT; 479 amino acid residues; ~ 53.54 kDa), *gadA* gene was inserted into an in-house vector pRSETA-SUMO with both 6×His tag (~ 0.84 kDa) and SUMO-protein tag (~ 12 kDa) at the N-terminus (refer to Table 1 for restriction sites selection and primers), 6×his-SUMO-tagged GadA (WT) was purified by Ni-NTA column and cleaved by SUMO protease, no extra residues were retained to its N-terminus. For *Lb. brevis* GadA (Δ5; 474 residues; ~ 52.94 kDa), the first five residues (MNKND; ~ 0.62 kDa) from the N-terminus of *Lb. brevis* GadA (WT) was removed during expression vector construction using vector pRSETA-SUMO (refer to Table 1 for restriction sites selection and primers), 6×his-SUMO-tagged GadA (Δ5) was also purified by Ni-NTA column and cleaved by SUMO protease, no extra residues were retained to its N-terminus. For the expression and purification of *Lb. brevis* GadA (+GSHM; 483 residues; ~ 53.96 kDa), *Lb. brevis* GadB (+GSHM; 472 residues, ~ 53.97 kDa) and *Lb. plantarum* GadB (+GSHM; 473 residues; ~ 53.99 kDa), each *gad* gene was inserted into the typical expression vector pET-28a(+) with the 6×His tag (~ 0.84 kDa) at the N-terminus (refer to Table 1 for restriction sites selection and primers), 6×his-tagged Gad was purified by Ni-NTA column and cleaved by thrombin, but 4 residues (GSHM; ~ 0.43 kDa) were retained in the N-terminus of these three Gads. The proteins were separated and stained in 10% SDS-PAGE gel. All target protein bands were further excised and identified by mass spectrometry (data not shown). Denotation: M, protein marker; S, supernatants of cytosolic proteins from *E. coli* after sonication; N, 6×his-SUMO-tagged protein (left panel) or 6×his-tagged protein (right panel) purified by Ni-NTA column; P, purification via gel filtration after cleavage of 6×his tag or/and 6×his-SUMO tag by thrombin or SUMO protease.
